# Impact of Age, Period, Cohort, Region, Race, and Health Services on Bladder Cancer Mortality in Brazil: A 23-Year Ecological Study

**DOI:** 10.3390/cancers16173038

**Published:** 2024-08-31

**Authors:** João Simão de Melo Neto, Sâmia Feitosa Miguez, Amanda Lia Rebelo Rabelo, Amanda Marinho da Silva, Daniel Souza Sacramento, Dária Barroso Serrão das Neves, Iana Nogueira Rego, Riter Lucas Miranda Garcia, Deizyane dos Reis Galhardo, André Luiz Machado das Neves

**Affiliations:** 1 Institute of Health Sciences, Federal University of Pará (UFPA), Belém 66075-110, PA, Brazil; deizyanegalhardo@gmail.com; 2 School of Health Sciences, University of the state of Amazonas (UEA), Manaus 69005-010, AM, Brazil; smiguez@uea.edu.br (S.F.M.); dsacramento.am@gmail.com (D.S.S.); dbneves@uea.edu.br (D.B.S.d.N.); almachado@uea.edu.br (A.L.M.d.N.); 3 Doctoral Program in Public Health in the Amazon, Federal University of Amazonas (UFAM), Manaus 69067-005, AM, Brazil; amandaliarr@gmail.com (A.L.R.R.); iana_nogueira@hotmail.com (I.N.R.); profriter@gmail.com (R.L.M.G.); 4 Leônidas and Maria Deane Institute, Oswaldo Cruz Foundation (FIOCRUZ), Manaus 69057-070, AM, Brazil; amandas.marinhos@gmail.com

**Keywords:** urinary bladder neoplasms, mortality registries, risk factors, epidemiology

## Abstract

**Simple Summary:**

Bladder cancer is an economically costly cancer, especially in Brazil, where treatment and diagnosis vary greatly across regions. This study revealed that, after the age of 50, mortality from this disease increases, and is more common among white people and residents of the southern region of Brazil. The lack of health professionals and the lower investment in health actions and services contributed to this increase in mortality. In addition, the performance of basic procedures, such as punctures and cystoscopy examinations, was lower in regions with higher mortality rates; however, more complex surgeries failed to reduce mortality rates. These results underscore the need to improve public health policies and ensure better access to healthcare for cancer, especially in Brazilian regions with fewer resources.

**Abstract:**

Bladder cancer is one of the most economically costly types of cancer, but few studies have evaluated its mortality considering the factors that impact this outcome. This study aimed to investigate the impact of sociodemographic factors, period, cohort, and health services on bladder cancer mortality. This ecological study analyzed bladder cancer mortality data in Brazil from 2000 to 2022 and evaluated sociodemographic variables (race, region of residence), socioeconomic variables (gross domestic product per capita, Gini index of household income per capita, number of health professionals per inhabitant, expenditure on public health services, and consultations per inhabitant), and bladder cancer diagnosis and treatment procedures. These data were subjected to statistical analysis, which revealed that after the age of 50, there was a progressive increase in the risk of bladder cancer. Indigenous people had the lowest mortality rate, while white people had a significantly greater mortality rate than black and brown people. The North Region and Northeast Region presented the lowest mortality rates, whereas the South Region presented the highest mortality rates. In the South and Southeast Regions, a higher GDP was related to lower mortality. In the South, higher mortality was associated with a lower number of consultations per inhabitant per region. Fewer bladder punctures/aspirations and bladder biopsies were associated with higher mortality rates. In oncology, more procedures, such as total cystectomy, cystoenteroplasty, and total cystectomy with a single shunt, do not reduce the mortality rate. These results can serve as guidelines for adjusting public health policies.

## 1. Introduction

Bladder cancer is one of the main types of cancer affecting the population globally [1]. The global incidence has been stable, but increases have been observed in certain regions of the world, impacting mortality, probably due to exposure to carcinogens [1]. In 2022, there were approximately 614,000 new cases and 220,000 deaths worldwide, representing approximately 3% of all new cases and more than 2% of cancer deaths [2].

Bladder cancer is one of the most economically costly types of cancer owing to the intensive treatment and monitoring needed, burdening patients and healthcare systems [3]. There are differences in survival and mortality rates between different regions of the world, which can be partly explained by differences in treatment protocols, healthcare systems, and access to diagnosis and treatment facilities [4]. In Brazil, there were an estimated 11,370 new cases and 5119 deaths in 2023. The estimated risk corresponds to 5.25 cases per 100,000 inhabitants, with men being more affected than women. These values represent an estimated risk of 7.45 new cases per 100,000 men [5]. However, few studies have evaluated mortality in the Brazilian population, and the studies found in this country refer to a specific region [6] and short periods without considering the country’s regional socioeconomic differences [7].

The most well-established risk factor for bladder cancer is smoking, which accounts for 30 to 50% of all cases. Other risk factors include age and sex, all of which have significant impacts on the development and progression of bladder cancer [8]. Thus, analyzing how these factors impact mortality in different administrative regions can contribute to the formulation of public policies.

In terms of race, the black population was associated with worse survival than other races. Although studies suggest a more aggressive manifestation of the disease in black patients, the disparity in mortality mostly reflects social barriers that disproportionately affect this population. These barriers include difficulties in accessing diagnosis and treatment, often resulting in the disease being diagnosed at an advanced stage [9]. In this sense, we believe that in Brazil, owing to the racial distribution in different regions, we can verify specific mortality rates.

Access to the public health system in Brazil may have improved from 2008 to 2017, as not only did the number of individuals increase significantly but also the proportion of cystectomies decreased, which may reflect an improvement in early-stage treatment for this type of cancer [7]. Nevertheless, the distribution of services is uneven across the country, and a more specific analysis is needed.

In this context, this study aimed to investigate the impact of sociodemographic factors (age, race, and region), period, cohort, and health services on bladder cancer mortality in Brazil from 2000 to 2022. Our hypothesis is that individuals over the age of 50 years in conditions of greater vulnerability (residents of the North and Northeast Regions; black and indigenous race) and regions with the lowest availability of health services have the highest mortality rates from bladder cancer.

## 2. Methods

### 2.1. Type of Study and Ethical Aspects

This is an observational study with an ecological design [10] that uses secondary data from public information systems on bladder cancer mortality in Brazil between 2000 and 2022. As the databases are in the public domain, without identifying the individuals, there was no need for approval by a Research Ethics Committee, as per the guidelines of the National Health Council of Brazil, CNS Resolution No. 510 (4 July 2016).

### 2.2. Population

Secondary data on all individuals who died from bladder cancer of both sexes were reviewed. The deaths analyzed were from the period between 2000 and 2022 and were registered in the Department of Informatics of the Unified Health System (DATASUS) of the Ministry of Health of Brazil (https://datasus.saude.gov.br/, accessed on 11 August 2024).

### 2.3. Inclusion and Exclusion Criteria

This study included patients who died between 2000 and 2022 and were classified under the ICD-10 code C67 (malignant neoplasm of the bladder) [11]. Individuals who died outside of the study period were excluded from the analysis, and individuals with unknown information were excluded from the analyses.

### 2.4. Database and Variables Analyzed

The variables investigated and their descriptions, the information systems consulted, and the collection period are listed in Table 1. The units of analysis were the five geographical regions of Brazil (North, Northeast, South, Southeast, and Midwest), according to the Brazilian Institute of Geography and Statistics.

The DATASUS of the Ministry of Health of Brazil offers a secondary open-access database. The mortality information used to calculate the age-standardized mortality rate (ASMR), as well as sociodemographic data, access factors, spending on public services, diagnosis, and treatment, were obtained from DATASUS through the records of the mortality information system (SIM), hospital information system (SIH), outpatient information system (SIA), and indicators and basic data (IDB).

The population data used to calculate the crude-specific mortality rate were found from a projection using population data from the Brazilian Institute of Geography and Statistics (IBGE) (https://ibge.gov.br/, accessed on 11 August 2024). The number of deaths used to calculate the age-adjusted standardized mortality rate was obtained according to place of residence. The world standard population according to the World Health Organization [12] was used to calculate the age-standardized mortality rate.

### 2.5. Statistical Analysis

To identify the influence of age, period, and birth cohort on deaths from bladder cancer, the APC Web tool (Biostatistics Branch, National Cancer Institute, Bethesda, MD, USA) was used [13]. The important functions of the APC offered by this tool are useful in oncological applications. As described in Nascimento et al. [14], the following parameters were studied in this model: net drift; all age, period, or cohort deviations; all period or cohort rate ratios (RRs); and all local drift. Additionally, Wald tests were applied followed by chi-square tests (x2) to identify statistically significant variables (*p* < 0.05) on the basis of age, period, and birth cohort. The age group of the study population and the number of deaths were grouped into 5-year intervals, resulting in 18 age groups (from 0–4 years to 85 years or more). In addition, 4 periods (2000–2004 to 2015–2019) and 21 birth cohorts were considered, each with an interval of 5 years (1915–2015).

The results of the descriptive analysis were expressed in measures of central tendency and dispersion. The normality of the data was evaluated with the Shapiro–Wilk test. The nonparametric Kruskal–Wallis test was used to verify whether there were statistically significant differences between the median crude mortality rates and those adjusted for race and geographic region.

A multivariate linear regression analysis was performed to assess the predictive value of the age-standardized mortality rate via the adjusted model.

The Durbin-Watson test was performed to verify the independence of the residuals, with an acceptable range of [1.5; 2.5]. A Cook’s distance below 1 meant that there were no outliers in the dataset that could impair the estimation of the coefficients. The variance inflation factor (VIF) (less than 10) and tolerance (greater than 0.2) values of the final model revealed the absence of multicollinearity.

In addition, we analyzed the Gaussian distribution and the P-P plot, in which a comparison between the “observed” and “expected” probabilities was used to test the normal distribution. We used the graph of “standardized residuals” versus “standardized predicted values” to verify the constancy of the variance of the residuals.

We conducted intergroup comparisons and multivariate linear regression analysis using the Statistical Package for Social Sciences (SPSS for Windows, version 21.0; IBM, Armonk, NY, USA), considering *p*-values less than 0.05 as statistically significant.

## 3. Results

We identified 78,015 bladder cancer deaths in Brazil from 2000 to 2022, with males accounting for 68.78% of these cases. Table 2 provides a descriptive analysis of the entire cohort.

### 3.1. Age–Period–Cohort Effect

**Figure 1:**
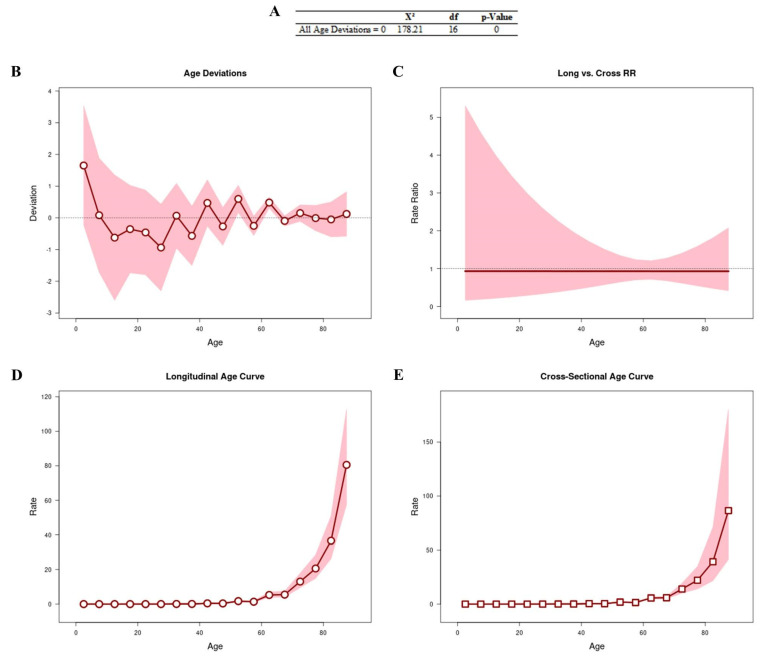
APC analysis to assess whether age is a determining factor in the number of deaths. All age deviations are shown in (**A**,**B**). The age curves are log-linear (**C**). Longitudinal (**D**) and transverse (**E**) age curves.

Figure 1 shows the results obtained from the APC analysis to assess whether age is a determining factor in the number of deaths. All age deviations (Figure 1A,B) showed that the adjusted longitudinal and transverse age curves were log-linear (Figure 1C). We observed that the age curves (longitudinal [RR: 1.74; 95% CI: 1.39–2.16] and cross-sectional [RR: 1.86; 95% CI: 1.32–2.61]) were linear, with an increased risk of progression after the age of 50 years (Figure 1D and Figure 1E, respectively).

**Figure 2:**
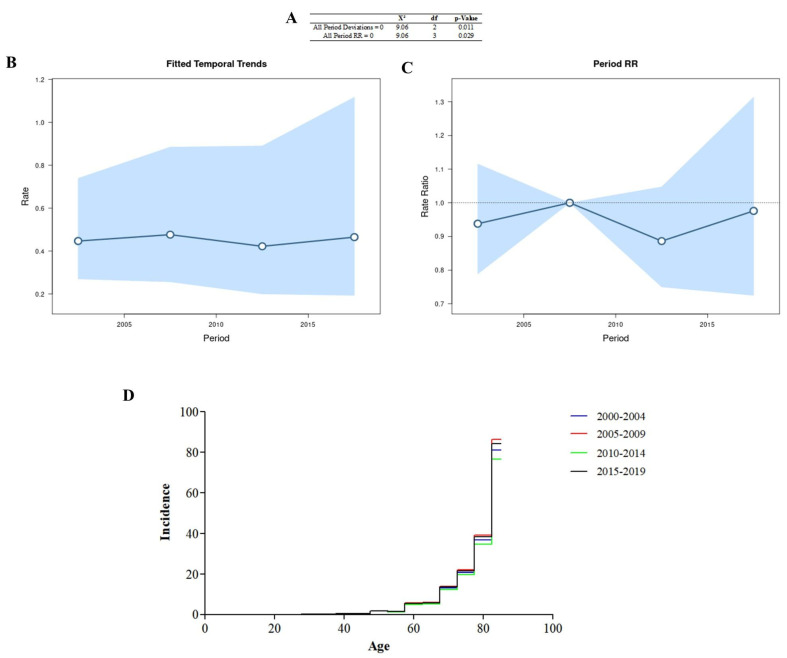
Age–period–cohort analysis was performed via the Wald test (**A**), temporal trends (**B**), period rate ratios (**C**), and the age incidence pattern for every period (**D**).

All period deviations were significant (Figure 2A), indicating that the adjusted time trends (Figure 2B) and period rates (Figure 2C) were log-linear. All period rate ratios were significant, and the age incidence pattern in each period (Figure 2D) was determined using the cross-sectional age curve (Figure 1E). The period 2005–2009 had the highest number of cases in the different years.

The values for the rate (χ^2^ = 30.78; df = 20; *p* = 0.058) and deviation (χ^2^ =18.35; df = 19; *p* = 0.499) for the cohort were not significant.

### 3.2. Sociodemographic Factors

Table 3 presents the socioeconomic predictors of bladder cancer mortality in the different administrative regions of Brazil. We observed that in the South Region and Southeast Region, a higher GDP was related to lower mortality.

**Figure 3:**
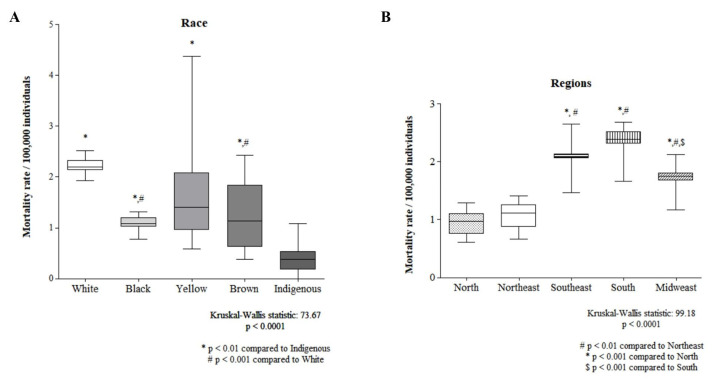
A comparison between races (**A**) and regions (**B**) is shown.

Figure 3 shows the comparison of mortality rates by race and regions of Brazil. The indigenous population had the lowest mortality rate, while that of the white population was significantly greater than that of the black and brown populations (Figure 3A). The North Region and Northeast Region presented the lowest mortality rates, whereas the South Region presented the highest mortality rate (Figure 3B).

### 3.3. Health Services, Diagnosis, and Treatment

Table 4 presents the access to and expenditures on public services and diagnoses that predict bladder cancer mortality in the different administrative regions of Brazil. In the Southeast Region, higher mortality was associated with a lower number of health professionals and lower expenses for public health actions and services. Nevertheless, in the South, higher mortality was associated with a lower number of consultations per inhabitant by region.

A lower rate of bladder puncture/aspiration was associated with higher mortality rates in Brazil, especially in the Northeast Region and South Region. Fewer cystoscopies and/or ureteroscopies and/or urethroscopies were associated with higher mortality in the North and Midwest Regions. In addition, fewer bladder biopsies were associated with higher death rates in Brazil, especially in the Midwest region.

Table 5 presents the treatments that predict bladder cancer mortality in the different administrative regions of Brazil. In the northern region, we found that less “cystostomy” was associated with a higher mortality rate. However, in oncology, more procedures, such as total cystectomy, cystoenteroplasty, and total cystectomy with a single shunt, do not reduce the mortality rate. A lower percentage of patients who underwent total cystectomy was related to a higher mortality rate in the Southeast Region.

## 4. Discussion

Bladder cancer is one of the most common neoplasms and is associated with high morbidity and mortality for patients [6], in addition to having a high cost of diagnosis and treatment [3]. In Brazil, regional disparities contribute to different patterns of mortality distribution [15]. In this study, we observed that age, period, birth cohort, sociodemographic factors, and those related to health services, diagnosis, and treatment in the different administrative regions of Brazil impacted the determinants of bladder cancer mortality between 2000 and 2022.

Men were the most affected during the evaluated period, representing approximately 70% of the deaths. Another associated factor was age, with a progressive increase in risk from the age of 50 in all regions of Brazil, corroborating the findings of other studies [7] that have also identified these factors. In addition, the number of hospital admissions after the age of 60 has been shown to be high in Brazil [15]. However, the age range found in our study differed from that reported in other studies, which indicated a predominance from the age of 60 years [6,16].

In 2008, the Ministry of Health established the National Policy for Comprehensive Care for Men’s Health, developed in partnership with managers of the Unified Health System and Scientific Society, with the aim of promoting health actions that increase life expectancy and reduce morbidity and mortality from preventable and preventable causes in this population, such as bladder cancer [17]. This may explain why the period from 2005 to 2009 had the highest number of cases, followed by a positive effect of this policy in subsequent years.

In the comparison between races and regions, indigenous people had the lowest mortality rate, whereas whites had significantly higher rates than blacks and browns. The lower mortality rate of indigenous people may be associated with lower exposure to risk factors for occupational exposure, such as aluminum production, rubber production, painting with artificial dyes, such as magenta, or environmental exposure to radiation, medications (cyclophosphamide), opium consumption, and *Schistosoma* infection [1], which are common in urban centers and are part of the daily life of Caucasian individuals; in another study, most of the men were white [7]. Another point to be highlighted is the barriers encountered by indigenous people in health services and high-cost diagnostic tests, which may have contributed to a lower detection of cases in this population [18].

In our study, the North and Northeast Regions had the lowest mortality rates, whereas the South Region presented the highest mortality rate. This finding converges with other studies that reported a 76% higher mortality rate and a 146% higher hospitalization rate in the South Region than the national average [6]. This phenomenon may be influenced by the cultural habit of consuming chimarrão, a drink based on Yerba mate, since it is associated with regular tobacco consumption [6], which is the greatest predictor of incidence and mortality from bladder cancer [8].

We observed that in the South Region and Southeast Region, a higher GDP was related to lower mortality. Studies have shown that countries with decreasing trends in bladder cancer mortality have a greater average GDP per capita than those with increasing trends [8]. A higher GDP may be associated with better eating habits, greater awareness of the disease, and better access to health services.

The results regarding health services, diagnosis, and treatment were alarming, showing that the lack of access to health services is associated with higher mortality in all the Federative regions of Brazil in different dimensions. In the Southeast Region, higher mortality was associated with a lower number of health professionals and lower expenditures on actions and public services. In the South, higher mortality is associated with a lower number of consultations per inhabitant, indicating that insufficient access to regular medical care can lead to late diagnoses and less effective treatments [19].

In addition, fewer procedures, such as bladder puncture/aspiration (Northeast and South), cystoscopy (North and Midwest), bladder biopsy (Midwest), and cystostomy (North), were associated with higher mortality rates. Although these procedures are often used only for diagnosis or prior treatment, their unavailability or delay in being performed can delay the start of definitive treatment, leading to the progression of the disease at the cost of a worsening prognosis [6].

Despite the fact that more procedures, such as total cystectomy (with or without a single shunt) and cystoenteroplasty, have been performed in the northern region than in the southern region, these procedures have not reduced the mortality rate, which reinforces the slowness of diagnosis and treatment since these procedures are performed in more serious cases and emergencies, possibly with a late diagnosis. Although mortality rates are higher for procedures such as these than for endoscopic procedures [7], it is important to take into account that total cystectomy offers an acceptable rate of perioperative complications and can be offered as a viable treatment option, even in elderly patients [20]. For the cystoenteroplasty procedure, since it involves reconstruction of the bladder via laparoscopy [21], these reconstruction procedures may be indicated in elderly patients if the patient’s condition is favorable [22].

The limitations of our study are that the use of secondary databases introduces an ecological bias since the analyses were carried out at the population level and not at the individual level. In addition, the quality of the data from these databases can vary significantly, impacting mortality estimates. The absence of outpatient and hospitalization data from health services not covered by the Brazilian Unified Health System also limits the scope of the results. There is the possibility of delays or errors when entering data into the Ministry of Health’s system, mainly due to disparities in connectivity infrastructure in the different regions of the country. Another important limitation is racial classification, which is determined by the medical team responsible for completing the death certificate, increasing the possibility of divergence in self-reported data. In addition, the nature of ecological studies prevents direct inferences of causality, and it is essential to carry out case–control or cohort studies.

## 5. Conclusions

This study revealed that there was an increase in the mortality rate from this condition after the age of 50 years in the period 2005–2009, which was more prevalent among white people and those living in the southern region of Brazil. A lower number of health professionals per inhabitant per region and lower spending on health actions and services had an impact on increasing mortality. A lower performance of “bladder puncture/aspiration”, “cystoscopy and/or ureteroscopy, and/or urethroscopy” and “cystostomy” was associated with higher mortality rates. However, the use of “total cystectomy”, “cystoenteroplasty”, or “total cystectomy with a single shunt in oncology” did not reduce the mortality rate.

## Figures and Tables

**Table 1:** Description of the variables analyzed, information systems consulted, and collection period.

Variables	Descriptions	Information Systems Consulted	Collection Period
**Dependent**			
Age-adjusted atandardized mortality ratio	Rate resulting from the application of crude bladder cancer mortality rates standardized by the world standard population and adjusted by age group (expressed in the number of deaths per 100,000 inhabitants).	SIM	2000–2022
**Independent**			
*Socioeconomic factors*			
Region of residence	Refers to the place of residence according to the territorial division of Brazil with particular characteristics. Categories: North, Northeast, Midwest, Southeast, and South.	SIM	2000–2022
Race	Refers to the classification of the population according to characteristics related to ethnic and racial origin. Categories: white, black, brown, yellow, and indigenous.	SIM	2000–2022
Gross domestic product (GDP) per capita	Percentage generated by dividing the GDP by the number of inhabitants in the region and measures how much of the GDP would go to each individual in a country if they all received equal shares.	IDB	2000–2010
Gini index of per capita household income	A measure of the degree of concentration of the distribution of household income per capita of a given population and in a given geographical space.	IDB	2000–2012
*Access factors and expenditure on public services*			
Number of health professionals per inhabitant	The number of health professionals in activity per thousand inhabitants according to categories in a given geographical area in the year considered.	IDB	2000–2010
Expenditure on public health actions and services as a proportion of the GDP	Percentage of the national GDP that corresponds to federal public spending on health in the year considered.	IDB	2000–2010
Consultations per inhabitants by region according to year	Number of medical consultations in the Unified Health System per inhabitant per year according to regions.	IDB	2000–2012
*Diagnosis*			
Urinary tract ultrasound (0205020054)	The number of noninvasive imaging tests used in the evaluation of the organs of the urinary system (kidneys, ureters, urethra, bladder, and prostate).	SIA	2008–2022
Lower abdomen CT scan (0206030037)	The number of imaging exams that, through axial cuts, provide a detailed study of different structures of the human body, facilitate localization, detect very small alterations in tissues, organs, and other structures of the lower abdomen, and provide greater precision in clinical and surgical interventions.	SIA	2008–2022
Lower abdominal and pelvic magnetic resonance imaging (MRI) (0207030022)	The number of magnetic resonance imaging exams that combine several image sequences, including T1-weighted images, T2-weighted images, and diffusion-weighted images (dwi), among others, to provide detailed information about the lower abdomen in order to confirm the suspicion of cancer.	SIA	2008–2022
Relief bladder catheterization (0301100047)	The number of sterile procedures that consist of inserting a tube into the bladder through the urethra in order to drain urine, which is removed once the purpose of the procedure has been achieved.	SIA	2008–2022
Delay bladder catheterization (0301100055)	The number of sterile procedures of a sterile catheter in the bladder through the urethra, with the aim of draining urine in situations of bladder incompetence and urinary incontinence.	SIA	2008–2022
Suprapubic aspiration (0409010359)	The number of procedures where a needle is inserted into the bladder through the anterior abdominal wall—crudely—to access the organ in order to collect or introduce substances for diagnostic or therapeutic purposes or drainage.	SIA	2008–2022
Cystoscopy and/or ureteroscopy and/or urethroscopy (0209020016)	The number of endoscopic examinations, without cuts, simple, and with low risks, carried out in patients with suspected diseases of the bladder or urethra (urine channel). They can be performed with rigid and flexible apparatus, with or without sedation.	SIA	2008–2022
Bladder biopsy (0201010062)	The number of procedures in which a tissue sample is removed and referred for analysis to a pathologist, a doctor who specializes in diagnosing diseases by analyzing tissues with a microscope.	SIA	2008–2022
*Treatment*			
Cystostomy (0304040070)	The number of urinary diversion surgeries, which make it possible to drain the bladder by the supra-pubic route, in situations where the urethra does not allow its emptying.	SIA	2008–2022
Chemotherapy for bladder cancer (0304040070)	The number of chemotherapies consisting of the use of anticancer drugs to destroy tumor cells in the bladder.	SIA	2008–2022
Endoscopic resection of bladder lesions (0409010383)	The number of surgeries in which the device is introduced through the urethra, under anesthesia, to inspect the bladder and remove the tumor in fragments for biopsy.	SIH	2008–2022
Partial cystectomy (040910022)	The number of surgeries of high complexity used to remove malignant tumors in the affected organ, when the function and structure of the organ can be partially preserved.	SIH	2008–2022
Total cystectomy (0409010030)	The number of highly complex surgeries used to remove the organ affected by malignant tumors.	SIH	2008–2022
Cystoenteroplasty (0409010057)	The number of bladder reconstruction surgeries with bowel loops after bladder cancer surgery.	SIH	2008–2022
Cystectomy with a shunt in only 1 time in oncology (0416010024)	The number of complete or partial resections of the bladder affected by malignant tumors, with or without ureteroenterostomy, in the case of a simple shunt with or without nephrostomy or ureterostomy.	SIH	2008–2022

Mortality information system (SIM); indicators and basic data (IDB); Ambulatory Information System (SIA); Hospital Information System of the Unified Health System (SIH).

**Table 2:** Descriptive analysis of the entire cohort.

	Brazil n = 78,015 (%)		North n = 2261 (%)		Northeast n = 12,392 (%)		Southeast n = 42,126 (%)		South n = 16,627 (%)		Midwest n = 4609 (%)	
**Sex**												
Male	53,658	68.78	1569	69.40	8147	65.75	28,925	68.66	11,884	71.47	3133	67.98
Female	24,350	31.21	692	30.60	4245	24.25	13,194	31.32	4743	28.53	1476	32.02
Unknown	7	0.008	0	0.00	0	0.00	7	0.02	0	0.00	0	0.00
**Race**												
White	53,382	68.42	717	31.71	4334	34.97	31,010	73.61	14,783	88.90	2538	55.06
Black	3975	5.09	103	4.55	899	7.25	2286	5.42	442	2.65	245	5.31
Yellow	582	0.74	10	0.44	32	0.25	438	1.03	71	0.42	31	0.67
Brown	16,497	21.14	1350	59.70	6280	50.67	6499	15.42	772	4.64	1596	34.62
Indigenous	62	0.07	11	0.48	17	0.13	13	0.03	7	0.04	14	0.30
Unknown	3517	4.50	70	3.09	830	6.69	1880	4.46	552	3.31	185	4.01
**Age (years)**												
≤29	214	0.27	24	1.06	58	0.46	82	0.19	29	0.17	21	0.45
30 to 39	501	0.64	31	1.37	143	1.15	205	0.48	84	0.50	38	0.82
40 to 49	2084	2.67	109	4.82	455	3.67	958	2.27	412	2.47	150	3.25
50 to 59	7221	9.25	271	11.98	1224	9.87	3733	8.86	1546	9.29	450	9.76
60 to 69	16,603	21.28	483	21.36	2553	20.59	9029	21.43	3568	21.45	970	21.04
≥70	51,371	65.84	1342	59.35	7953	64.16	28,109	66.72	10,987	66.07	2980	64.65
Unknown	21	0.02	1	0.04	9	0.07	10	0.02	1	0.006	0	0.00

**Table 3:** Socioeconomic predictors of bladder cancer mortality in the different administrative regions of Brazil.

		North	Northeast	Southeast	South	Midwest	Brazil
Gross domestic product (GDP) per capita	β	−0.110	0.357	−0.735	−0.907	−0.296	−0.532
	t	−0.333	1.148	−3.248	−3.618	−0.929	−1.884
	*p*	0.747	0.280	0.010 *	0.007 *	0.377	0.092
Gini index	β	−0.079	−0.434	−0.141	−0.361	−0.343	−0.400
	t	−0.222	−1.363	−0.545	−1.440	−0.951	−1.306
	*p*	0.830	0.210	0.601	0.188	0.369	0.228

* *p* < 0.05 indicates a significant difference.

**Table 4:** Access to and expenditure on public services and diagnoses that predict bladder cancer mortality in the different administrative regions of Brazil.

		North	Northeast	Southeast	South	Midwest	Brazil
**Health services**							
Number of health professionals per inhabitant	β	0.026	0.585	−0.682	−0.087	−0.237	-
	t	0.075	0.807	−13.208	−0.327	−0.510	-
	*p*	0.942	0.446	<0.0001 *	0.753	0.626	-
Expenses for public health actions and services as a proportion of GDP	β	−0.445	0.575	−0.652	−0.034	0.479	−0.055
	t	−1.287	2.110	−12.635	−0.123	1.123	−0.131
	*p*	0.234	0.064	<0.0001 *	0.906	0.298	0.899
Consultations per inhabitant by region	β	−0.565	−0.024	−0.577	−0.722	−0.207	−0.355
	t	−0.685	−0.047	−1.278	−2.733	−0.539	−0.852
	*p*	0.515	0.964	0.242	0.029 *	0.607	0.419
**Diagnostic**							
Urinary tract ultrasound	β	0.053	-	−0.578	-	0.391	−0.154
	t	0.200	-	−1.137	-	0.876	0.416
	*p*	0.845	-	0.285	-	0.415	0.687
Tomography of the lower abdomen	β	-	0.341	0.338	-	−0.874	-
	t	-	1.864	0.657	-	−1.404	-
	*p*	-	0.092	0.528	-	0.210	-
MRI of pelvis and pelvis/lower abdomen	β	0.107	-	-	−0.273	-	−0.479
	t	0.604	-	-	−1.135	-	−1.411
	*p*	0.559	-	-	0.283	-	0.192
Bladder puncture/aspiration	β	-	−0.643	−0.526	−0.980	−0.516	−2.134
	t	-	−2.930	−0.820	−4.170	−0.923	−5.933
	*p*	-	0.015 *	0.433	0.002 *	0.391	<0.0001 *
Cystoscopy and/or ureteroscopy and/or urethroscopy	β	−0.563	0.066	0.604	−0.016	1.517	0.247
	t	−2.448	0.377	1.241	−0.087	2.504	0.776
	*p*	0.034 *	0.714	0.246	0.933	0.046 *	0.457
Bladder biopsy	β	−0.366	−0.065	0.691	0.064	−1.777	1.266
	t	−1.265	−0.390	1.203	0.359	−2.988	4.567
	*p*	0.235	0.705	0.260	0.727	0.024 *	0.001 *

* *p* < 0.05 represents a significant difference. GDP—gross domestic product.

**Table 5:** Treatment that predicts bladder cancer mortality in the different administrative regions of Brazil.

		North	Northeast	Southeast	South	Midwest	Brazil
Cystostomy	β	−1.223	−0.349	0.080	−0.106	−0.104	−0.391
	t	−6.385	−0.816	0.330	−0.141	−0.158	−0.571
	*p*	0.001 *	0.452	0.753	0.893	0.881	0.589
Chemotherapy for bladder carcinoma	β	-	0.942	-	0.142	−0.736	-
	t	-	2.329	-	0.124	−1.209	-
	*p*	-	0.067	-	0.906	0.281	-
Bladder catheterization	β	0.026	−0.349	0.225	0.263	0.138	−0.087
	t	0.199	−0.816	1.639	0.465	0.444	−0.217
	*p*	0.849	0.452	0.152	0.661	0.676	0.835
Endoscopic resection of bladder lesions	β	0.248	−0.295	−0.359	0.516	0.764	0.079
	t	1.797	−0.751	−1.452	0.513	1.452	0.174
	*p*	0.122	0.486	0.197	0.630	0.206	0.868
Partial cystectomy	β	0.026	0.209	0.101	0.171	0.294	−0.345
	t	0.127	0.768	0.462	0.191	0.844	−1.190
	*p*	0.903	0.477	0.660	0.856	0.437	0.279
Total cystectomy	β	0.646	0.032	−1.215	0.505	−0.027	−0.520
	t	3.612	0.118	−4.948	0.720	−0.097	−0.765
	*p*	0.011 *	0.911	0.003 *	0.504	0.927	0.473
Cystoenteroplasty	β	0.386	0.200	0.015	−0.250	0.537	0.057
	t	2.665	0.617	0.098	−0.222	1.031	0.136
	*p*	0.037 *	0.564	0.925	0.833	0.350	0.896
Cystectomy with a single shunt in oncology	β	0.239	−0.068	0.042	0.289	−0.977	0.720
	t	1.615	−0.211	0.251	0.328	−1.644	1.647
	*p*	0.157	0.841	0.810	0.757	0.161	0.151
Total cystectomy with a single shunt in oncology	β	0.558	0.010	0.032	−0.029	−0.465	0.425
	t	4.752	0.038	0.193	−0.041	−1.373	1.040
	*p*	0.003 *	0.971	0.853	0.969	0.228	0.339

* *p* < 0.05 represents a significant difference.

## Data Availability

All data were made available at https://opendatasus.saude.gov.br/dataset, accessed on 11 August 2024.
